# Development of Novel ^123^I-Labeled Pyridyl Benzofuran Derivatives for SPECT Imaging of β-Amyloid Plaques in Alzheimer’s Disease

**DOI:** 10.1371/journal.pone.0074104

**Published:** 2013-09-13

**Authors:** Masahiro Ono, Yan Cheng, Hiroyuki Kimura, Hiroyuki Watanabe, Kenji Matsumura, Masashi Yoshimura, Shimpei Iikuni, Yoko Okamoto, Masafumi Ihara, Ryosuke Takahashi, Hideo Saji

**Affiliations:** 1 Department of Patho-Functional Bioanalysis, Graduate School of Pharmaceutical Sciences, Kyoto University, Kyoto, Japan; 2 Department of Neuroscience, Graduate School of Medicine, Kyoto University, Kyoto, Japan; NIH, United States of America

## Abstract

Imaging of β-amyloid (Aβ) plaques in the brain may facilitate the diagnosis of cerebral β-amyloidosis, risk prediction of Alzheimer’s disease (AD), and effectiveness of anti-amyloid therapies. The purpose of this study was to evaluate novel ^123^I-labeled pyridyl benzofuran derivatives as SPECT probes for Aβ imaging. The formation of a pyridyl benzofuran backbone was accomplished by Suzuki coupling. [^123^I/^125^I]-labeled pyridyl benzofuran derivatives were readily prepared by an iododestannylation reaction. In vitro Aβ binding assays were carried out using Aβ(1–42) aggregates and postmortem human brain sections. Biodistribution experiments were conducted in normal mice at 2, 10, 30, and 60 min postinjection. Aβ labeling in vivo was evaluated by small-animal SPECT/CT in Tg2576 transgenic mice injected with [^123^I]**8**. Ex vivo autoradiography of the brain sections was performed after SPECT/CT. Iodinated pyridyl benzofuran derivatives showed excellent affinity for Aβ(1–42) aggregates (2.4 to 10.3 nM) and intensely labeled Aβ plaques in autoradiographs of postmortem AD brain sections. In biodistribution experiments using normal mice, all these derivatives displayed high initial uptake (4.03–5.49% ID/g at 10 min). [^125^I]**8** displayed the quickest clearance from the brain (1.30% ID/g at 60 min). SPECT/CT with [^123^I]**8** revealed higher uptake of radioactivity in the Tg2576 mouse brain than the wild-type mouse brain. Ex vivo autoradiography showed in vivo binding of [^123^I]**8** to Aβ plaques in the Tg2576 mouse brain. These combined results warrant further investigation of [^123^I]**8** as a SPECT imaging agent for visualizing Aβ plaques in the AD brain.

## Introduction

Alzheimer’s disease (AD) is a leading cause of dementia with symptoms that include cognitive decline, irreversible memory loss, disorientation, language impairment, and an inability to carry out normal daily functions. Currently, it is difficult for clinicians to differentiate between the cognitive decline associated with normal aging and that associated with AD. A definitive diagnosis of AD can only be established by postmortem histopathological examination. Postmortem brains of AD patients reveal neuropathological features including senile plaques (SPs) and neurofibrillary tangles (NFTs), which contain β-amyloid (Aβ) peptides and highly phospholylated tau proteins, respectively [1], [2]. In particular, the formation of Aβ plaques in the brain has been proposed to play an important role in the pathogenesis of AD for several reasons: 1) Aβ deposits years before the onset of AD and can precede NFT formation; 2) mutation in the APP gene leads to some forms of early onset familial AD; and 3) Aβ is toxic to cultured neurons [3]–[5]. The availability of probes for imaging Aβ in vivo with noninvasive techniques such as positron emission tomography (PET) and single photon emission computed tomography (SPECT) would enable the presymptomatic diagnosis of cerebral β-amyloidosis, risk prediction of AD, and monitoring of the effectiveness of anti-amyloid therapies [4]. Thus, great efforts have been made to develop radiotracers that bind to Aβ plaques in vivo [6]–[10].

Over the past few years, initial studies with PET have suggested that many radiotracers show affinity for Aβ in vivo, several of which have been subjected to clinical trials, including [^11^C]2-(4′-(methylaminophenyl)-6-hydroxybenzothiazole (PIB) [11], [12], 2-(3-[^18^F]-fluoro-4-methyaminophenyl)benzothiazol-6-ol (GE-067, flutemetamol) [13], (*E*)-4-(*N*-methylamino)-4′-(2-(2-(2-[^18^F]-fluoroethoxy)ethoxy)ethoxy)-stilbene (BAY94-9172, florbetaben) [14], (*E*)-4-(2-(6-(2-(2-(2-[^18^F]fluoroethoxy)ethoxy)ethoxy)pyridin-3-yl)vinyl)-*N*-methylaniline (AV-45, florbetapir) [15]–[17], and 2-(2-[^18^F]-fluoro-6-(methylamino)pyridin-3-yl)benzofuran-5-ol (AZD4694) [18]. AV-45 has recently been approved by the US Food and Drug Administration (FDA) for the clinical diagnosis of AD by exclusion. These PET probes differ in their uptake and retention in the brain between AD patients and controls, suggesting the feasibility of visualizing Aβ plaques in AD brains. However, the short half-lives of C-11 (20 min) and F-18 (110 min) mean they must be prepared from a cyclotron on-site, and the number of facilities with PET cameras is limited. PET is similar to SPECT in its use of radiolabeled probes and detection of γ rays. In contrast with PET, however, the probes used in SPECT emit γ radiation that is measured directly, whereas PET probes emit positrons that annihilate electrons, causing two γ photons to be emitted in opposite directions. A PET scanner detects these emissions coincidentally to provide higher resolution images than SPECT. Conversely, SPECT scans are significantly less expensive than PET scans because they are able to utilize longer-lived more easily-obtained radioisotopes such as ^99m^Tc (6 h) or ^123^I (13 h) than PET. Then, since SPECT would be more appropriate than PET for routine diagnostic use, there is a need to develop more useful Aβ imaging agents for SPECT to diagnose preclinical AD patients, the numbers of which are expected to increase as populations continue to age rapidly all over the world.

Although several radioiodinated or ^99m^Tc-labeled ligands for Aβ plaques have been reported, unfavorable pharmacokinetics in vivo such as low uptake into the brain and slow washout have prevented clinical studies [9]. [^123^I]6-iodo-2-(4′-dimethylamino)phenyl-imidazo[1,2-*a*]pyridine (IMPY) is the first SPECT imaging agent to be tested in humans [19]–[21]. Although [^123^I]IMPY showed excellent properties as a Aβ imaging probe in preclinical studies, the preliminary clinical data displayed a low signal-to noise ratio, making it difficult to distinguish AD patients, possibly due to high lipophilicity and low stability in vivo [21]. There is therefore no SPECT imaging probe useful for the clinical diagnosis of AD.

Recently, we have reported the utility of a series of novel ^18^F-labeled benzofuran derivatives for PET imaging of Aβ plaques in the brain [22]–[24]. One of the derivatives, [^18^F]FPYBF-2, showed selective affinity for Aβ plaques and good pharmacokinetics in the brain, and is currently in preparation for clinical trials [24]. More recently, we have reported the development of ^99m^Tc-labeled pyridyl benzofuran derivatives as SPECT probes targeting Aβ plaques [25]. Although the ^99m^Tc derivative successfully labeled some Aβ plaques in ex vivo autoradiographic studies, there were many Aβ plaques that could not be labeled, indicating that further improvement of the binding affinity to Aβ aggregates and brain uptake is necessary. This may be attributable to the bulkiness of the ^99m^Tc-complex introduced in the pyridyl benzofuran scaffold. To develop more promising radiotracers for SPECT-based imaging of Aβ plaques, we planned to change ^99m^Tc to ^123^I as the radioisotope introduced in the pyridyl benzofuran scaffold.

In the present study, we designed and synthesized a novel series of iodinated pyridyl benzofuran derivatives and evaluated their biological potential as SPECT probes for visualizing Aβ plaques in vivo. Herein, we report the in vitro and in vivo evaluation and SPECT/CT imaging of these derivatives.

## Materials and Methods

### General

All reagents used in syntheses were purchased from Nacalai Tesque, Inc., Wako Pure Chemical Industries, Ltd., or Aldrich Co., and were used without further purification. ^1^H-NMR spectra were obtained at 400 MHz on JEOL JNM-AL400 NMR spectrometers at room temperature with tetramethylsilane (TMS) as an internal standard. Chemical shifts are reported as δ values (parts per million) relative to the internal TMS. Coupling constants are reported in Hertz. Multiplicity is defined by s (singlet), d (doublet), t (triplet), and m (multiplet). Mass spectra were acquired with a Shimadzu GC-MS-QP2010 Plus (ESI). Fluorescence observations were made using a microscope (Nikon Eclipse 80i) with a BV-2A filter set (excitation, 400–440 nm; diachronic mirror, 455 nm; long pass filter, 470 nm). Reversed-phase HPLC (RP-HPLC) and analyses of radiolabeled and nonradioactive analogs were performed with a Shimadzu system (a LC-10AT pump with a SPD-10A UV detector, *λ* = 254 nm) and a Cosmosil C18 column (Nacalai Tesque, 5C_18_-AR-II, 4.6 mm × 150 mm) using a mobile phase (acetonitrile : water = 7∶ 3) delivered at a flow rate of 1.0 mL/min. ddY mice (5 weeks, 22–25 g, male) were purchased from Shimizu Laboratory Supplies Co. Ltd. Tg2576 transgenic mice and wild-type mice were purchased from Taconic Farms, Inc. Small-animal µSPECT/CT (FX3300; Gamma Medica-Ideas, Sherbrooke, Canada) was conducted in the Radioisotope Research Center, Kyoto University. All animal studies were conducted in accordance with our institutional guidelines and approved by Kyoto University Animal Care Committee.

### Chemistry

#### 5-(5-Bromobenzofuran-2-yl)pyridin-2-amine (1)

A solution of 5-bromobenzofuran-2-boronic acid (722 mg, 3.0 mmol), 2-amino-5-iodopyridine (660 mg, 3.0 mmol), and Pd(Ph_3_P)_4_ (366 mg, 0.3 mmol) in 2 M Na_2_CO_3_ (aq.)/dioxane (150 mL, 1∶1) was stirred under reflux overnight. The mixture was allowed to cool to room temperature, and 1 M NaOH (20 mL) was added. After extraction with ethyl acetate, the organic phase was dried over Na_2_SO_4_ and filtered. The filtrate was concentrated and the residue was purified by silica gel chromatography (hexane : ethyl acetate = 1∶ 6) to give 273 mg of **1** (31.5%). ^1^H NMR (400 MHz, CDCl_3_): δ 4.66 (s, 2H), 6.57 (d, 1H, *J = *8.4 Hz), 6.79 (s, 1H), 7.32 (d, 1H, *J* = 2.4 Hz), 7.33 (d, 1H, *J = *2.0 Hz), 7.66 (d, 1H, *J* = 1.6 Hz), 7.86 (dd, 1H, *J_1_* = 8.8 Hz, *J_2_* = 2.4 Hz), 8.58 (d, 1H, *J = *2.4 Hz). MS: m/z 290 (M^+^+H).

#### 5-(5-Bromobenzofuran-2-yl)-*N*-methylpyridin-2-amine (2)

The same reaction as described above to prepare **1** was employed, and **2** was obtained in 38.0% yield from 5-bromobenzofuran-2-boronic acid and 5-iodo-*N*-methylpyridin-2-amine. ^1^H NMR (400 MHz, CDCl_3_): δ 2.98 (d, 3H, *J* = 5.2 Hz), 4.80 (s, 1H), 6.45 (d, 1H, *J* = 8.8 Hz), 6.75 (s, 1H), 7.32 (d, 1H, *J* = 7.2 Hz), 7.52 (d, 1H, *J* = 8.4 Hz), 7.64 (d, 1H, *J* = 2.0 Hz), 7.87 (dd, 1H, *J_1_* = 8.8 Hz, *J_2_* = 2.4 Hz), 8.60 (d, 1H, *J* = 2.8 Hz). MS: m/z 304 (M^+^+H).

#### 5-(5-Bromobenzofuran-2-yl)-*N,N*-dimethylpyridin-2-amine (3)

The same reaction as described above to prepare **1** was employed, and **3** was obtained in 37.2% yield from 5-bromobenzofuran-2-boronic acid and 5-iodo-*N,N*-dimethylpyridin-2-amine. ^1^H NMR (400 MHz, CDCl_3_): δ 3.14 (s, 6H), 6.55 (d, 1H, *J* = 8.8 Hz), 6.71 (s, 1H), 7.17 (dd, 1H, *J_1_* = 8.8 Hz, *J_2_* = 2.4 Hz), 7.32 (d, 1H, *J* = 2.4 Hz), 7.53 (d, 1H, *J* = 8.8 Hz), 7.88 (dd, 1H, *J_1_* = 8.8 Hz, *J_2_* = 2.4 Hz), 8.65 (d, 1H, *J* = 2.4 Hz). MS: m/z 318 (M^+^+H).

#### 5-(5-(Tributylstannyl)benzofuran-2-yl)pyridin-2-amine (4)

A mixture of **1** (273 mg, 0.95 mmol), bis(tributyltin) (0.8 mL), and (Ph_3_P)_4_Pd (100 mg) in a mixed solvent (40 mL, 3∶1 dioxane/triethylamine mixture) was stirred at 90°C overnight. After extraction with ethyl acetate, the organic phase was dried over Na_2_SO_4_ and filtered. The filtrate was concentrated and the residue was purified by silica gel chromatography (hexane : ethyl acetate = 1∶ 1) to give 115.6 mg of **4** (24.6%). ^1^H NMR (400 MHz, CDCl_3_): δ 0.87–0.91 (m, 9H), 1.06–1.10 (m, 6H), 1.32–1.39 (m, 6H), 1.54–1.62 (m, 6H), 4.75 (s, 2H), 6.54 (d, 1H, *J* = 8.0 Hz), 6.82 (s, 1H), 7.31 (d, 1H, *J* = 8.0 Hz), 7.47 (d, 1H, *J* = 8.4 Hz), 7.63 (s, 1H), 7.85 (dd, 1H, *J_1_* = 8.4 Hz, *J_2_* = 2.4 Hz), 8.59 (d, 1H, *J* = 2.4 Hz). HRMS (EI): m/z calcd for C_25_H_36_N_2_OSn (M^+^) 500.1849, found 500.1847.

#### *N*-Methyl-5-(5-(tributylstannyl)benzofuran-2-yl)pyridin-2-amine (5)

The same reaction as described above to prepare **4** was used, and 27.7 mg of **5** was obtained in 28.4% yield from **2**. ^1^H NMR (400 MHz, CDCl_3_): δ 0.89 (t, 9H, *J* = 7.2 Hz), 1.32–1.35 (m, 12H), 1.62–1.66 (m, 6H), 3.04 (s, 3H), 6.75 (d, 1H, *J* = 8.8 Hz), 6.87 (s, 1H), 7.32 (d, 1H, *J* = 7.2 Hz), 7.46 (d, 1H, *J* = 8.4 Hz), 7.64 (d, 1H, *J* = 2.0 Hz), 8.05 (d, 1H, *J* = 8.4 Hz), 8.40 (d, 1H, *J* = 3.2 Hz). HRMS (EI): m/z calcd for C_26_H_38_N_2_OSn (M^+^) 514.2006, found 514.1998.

#### *N,N*-Dimethyl-5-(5-(tributylstannyl)benzofuran-2-yl)pyridin-2-amine (6)

The same reaction as described above to prepare **4** was used, and 88.9 mg of **6** was obtained in 25.1% yield from **3**.^ 1^H NMR (400 MHz, CDCl_3_): δ 0.89 (t, 9H, *J* = 7.2 Hz), 1.04–1.12 (m, 6H), 1.30–1.39 (m, 6H), 1.53–1.60 (m, 6H), 3.14 (s, 6H), 6.57 (d, 1H, *J* = 9.6 Hz), 6.79 (s, 1H), 7.29 (d, 1H, *J* = 8.0 Hz), 7.47 (d, 1H, *J* = 8.4 Hz), 7.62 (s, 1H), 7.88 (dd, 1H, *J_1_* = 9.6 Hz, *J_2_* = 2.4 Hz), 8.67 (d, 1H, *J* = 2.4 Hz). HRMS (EI): m/z calcd for C_27_H_40_N_2_OSn (M^+^) 528.2162, found 528.2158.

#### 5-(5-Iodobenzofuran-2-yl)pyridin-2-amine (7)

To a solution of **4** (100 mg, 0.20 mmol) in CHCl_3_ (5 mL) was added a solution of iodine in CHCl_3_ (0.5 mL, 1 M) at room temperature. The mixture was stirred at room temperature for 10 min. A NaHSO_3_ solution (3 mL, 5% in water) was added successively. The mixture was stirred for 5 min, and the organic phase was separated. The aqueous phase was extracted with CHCl_3_, and the combined organic phase was dried over Na_2_SO_4_ and filtered. The filtrate was concentrated and the residue was purified by silica gel chromatography (hexane : ethyl acetate = 1∶ 1) to give 34.9 mg of **7** (52.0%). ^1^H NMR (400 MHz, CDCl_3_): δ 4.66 (s, 2H), 6.57 (d, 1H, *J* = 8.4 Hz), 6.77 (s, 1H), 7.27 (s, 1H), 7.51 (d, 1H, *J* = 8.0 Hz), 7.84 (s, 1H), 7.85 (d, 1H, *J* = 2.4 Hz), 8.58 (d, 1H, *J* = 2.4 Hz). HRMS (EI): m/z calcd for C_13_H_9_IN_2_O (M^+^) 335.9760, found 335.9752.

#### 5-(5-Iodobenzofuran-2-yl)-*N*-methylpyridin-2-amine (8)

The same reaction as described above to prepare **7** was used, and 50.2 mg of **8** was obtained in 65.1% yield from **5**.^ 1^H NMR (400 MHz, CDCl_3_): δ 2.98 (d, 3H, *J* = 4.8 Hz), 4.79 (s, 1H), 6.45 (d, 1H, *J* = 8.8 Hz), 6.74 (s, 1H), 7.30 (s, 1H), 7.50 (d, 1H, *J* = 8.4 Hz), 7.85 (s, 1H), 7.87 (d, 1H, *J* = 2.4 Hz), 8.60 (d, 1H, *J* = 2.4 Hz). HRMS (EI): m/z calcd for C_14_H_11_IN_2_O (M^+^) 349.9916, found 349.9923.

#### 5-(5-Iodobenzofuran-2-yl)-*N,N*-dimethylpyridin-2-amine (9)

The same reaction as described above to prepare **7** was used, and 32.8 mg of **9** was obtained in 45.6% yield from **6**.^ 1^H NMR (400 MHz, CDCl_3_): δ 3.14 (s, 6H), 6.55 (d, 1H, *J* = 8.8 Hz), 6.70 (s, 1H), 7.25 (d, 1H, *J* = 4.0 Hz), 7.47 (d, 1H, *J* = 8.4 Hz), 7.82 (s, 1H), 7.84 (d, 1H, *J* = 2.4 Hz), 8.65 (d, 1H, *J* = 2.4 Hz). HRMS (EI): m/z calcd for C_15_H_13_IN_2_O (M^+^) 364.0073, found 364.0065.

### Radiosynthesis

Radioiodinated forms of pyridyl benzofuran derivatives were synthesized from the corresponding tributyltin derivatives. Briefly, to initiate the reaction, 100 µL H_2_O_2_ (3%) was added to a mixture of a tributyltin derivative (0.5 mg/100 µL of EtOH), 7.4 MBq of [^123^I]NaI or 0.74 MBq of [^125^I]NaI, and 100 µL of 1 N HCl in a sealed vial. The reaction was allowed to proceed at room temperature for 10 min and was terminated by the addition of NaHSO_3_. After neutralization with sodium bicarbonate, the reaction mixture was extracted with ethyl acetate. The extract was dried by passing through an anhydrous Na_2_SO_4_ column and blown dry with a stream of nitrogen gas. The radiolabeled ligands were purified by RP-HPLC.

### In Vitro Binding Assays Using Aggregated Aβ Peptides in Solution

Aβ(1–42) was purchased from the Peptide Institute (Osaka, Japan). Aggregation was carried out by gently dissolving the peptide (0.25 mg/mL) in a buffer solution (pH 7.4) containing 10 mM sodium phosphate and 1 mM EDTA. The solution was incubated at 37°C for 42 h with gentle and constant shaking. A mixture containing 50 µL of iodinated pyridyl benzofuran derivative (0.008 pM-400 µM in 10% EtOH), 50 µL of [^125^I]IMPY (0.02 nM), 50 µL of Aβ(1–42) aggregates, and 850 µL of 10% EtOH was incubated at room temperature for 3 h. The mixture was then filtered through Whatman GF/B filters using a Brandel M-24 cell harvester, and the radioactivity of the filters containing the bound ^125^I ligand was measured in a γ counter. Values for the half-maximal inhibitory concentration (IC_50_) were determined from displacement curves of three independent experiments using GraphPad Prism 5.0, and those for the inhibition constant (*K_i_*) were calculated using the Cheng-Prusoff equation [26]: *K_i_* = IC_50_/(1+ [L]/*K_d_*), where [L] is the concentration of [^125^I]IMPY used in the assay and *K_d_* is the dissociation constant of IMPY (4.2 nM).

### In Vitro Stability in Mouse Plasma

[^125^I]**8** (11 kBq, 10 mL) was added to the mouse plasma (200 mL), and the plasma samples were incubated at 37°C for 1 h. After incubation, plasma samples were mixed with equal volumes of acetonitrile followed by centrifugation at 4,500 rpm for 10 min to remove the denatured proteins. The supernatant was filtrated using a 0.45 µm filter (Millipore; Billerica, MA, USA). Then, the filtrate was analyzed by RP-HPLC.

### In Vivo Stability in Normal Mice

[^125^I]**8** (0.11 MBq, 100 µL) was injected into the tail vein of ddY mice. The mice were sacrificed at 2, 10, and 30 min after the injection (male, n = 3 for each time point). The blood samples were centrifuged at 3,500 rpm for 10 min to separate plasma. Plasma samples were mixed with equal volumes of acetonitrile followed by centrifugation at 4,500 rpm for 10 min to remove the denatured proteins. The supernatant was filtrated using 0.45 µm filter (Millipore; Billerica, MA, USA). Then, the filtrate was analyzed by RP-HPLC.

### Biodistribution in Normal Mice

While under anesthesia with 2% isoflurane, ddY mice were injected directly into a tail vein with 100 µL of 10% EtOH containing ^125^I-labeled pyridyl benzofuran derivatives (37 kBq). The mice (male, n = 5 for each time point) were sacrificed at 2, 10, 30, and 60 min postinjection. The organs of interest were removed and weighed, and radioactivity was measured with an automatic gamma counter (COBRAII; Packard). Percentage dose per organ was calculated by comparing the tissue counts to suitably diluted aliquots of the injected material. The %dose/g of samples was calculated by comparing the sample counts with the count of the diluted initial dose.

### Small-Animal SPECT/CT

Dynamic SPECT scans were performed on a dedicated small-animal SPECT scanner (FX3300, Gamma Medica-Ideas). Tg2576 transgenic mice (28 months, female, n = 3) and wild-type mice (28 months, female, n = 3) were used as an Alzheimer’s model and an age-matched control, respectively. Briefly, the animals were initially anesthetized with 2% isoflurane. When fully anesthetized, an animal was placed on the scanner bed, with a nose cone used to maintain anesthesia with 2% isoflurane throughout the experiment. [^123^I]**8** (20.5–26.5 MBq) in 10% aqueous EtOH solution containing 0.1% Tween80 was injected into a tail vein. Dynamic data were acquired over a period of 60 min after the injection, followed by the acquisition of CT data over a period of 5 min. The SPECT data were reconstructed using a 3-dimensional ordered subsets expectation maximization (3D OSEM) algorithm (5 iteration, 8 subsets). The SPECT scan time was sorted by a 30-s time frame in each 1-min interval (total, 60 frames for a total scan time of 60 min). Overexpression of Aβ plaques was observed in the cortex of Tg2576 mice, but not in the cerebellum of Tg2576 mice or in any brain regions of wild-type mice. For statistical analyses, regions of interest (ROIs) were manually placed over the frontal cortex (FR) and cerebellum (CE) on the summed SPECT images of [^123^I]**8** in Tg2576 (n = 3) and wild-type (n = 3) mice. Identification of anatomical structures was guided by the stereotaxic mouse brain atlas. Differences in FR to CE ratio values between Tg2576 and wild-type mice were compared by unpaired t tests. The level of statistical significance was designated as *p*<0.05.

### Ex Vivo Autoradiography of Transgenic Mouse Brain

The animals were killed by decapitation after the SPECT analysis. Brains were immediately removed and frozen in a dry ice/hexane bath. Sections of 20 µm were cut and exposed to a BAS imaging plate (Fuji Film, Tokyo, Japan) overnight. Ex vivo film autoradiograms were thus obtained. After autoradiographic examination, the same sections were stained by thioflavin-S to confirm the presence of Aβ plaques. For staining with thioflavin-S, sections were immersed in a 0.125% thioflavin-S solution containing 50% EtOH for 5 min and washed in 50% EtOH. After drying, the sections were examined using a microscope (Eclipse 80i; Nikon) equipped with a B-2A filter set (excitation, 450–490 nm; diachronic mirror, 505 nm; long-pass filter, 520 nm).

### In Vitro Autoradiography Using Human AD Brain Sections

Postmortem brain tissues from an anonymous autopsy-confirmed case of AD (93-year-old, female) were obtained from the Graduate School of Medicine, Kyoto University. Six-micrometer-thick serial sections of paraffin-embedded blocks were used for in vitro autoradiography. The sections were incubated with ^125^I-labeled tracers (444 kBq/50 µL) for 1 h at room temperature, then dipped in saturated Li_2_CO_3_ in 40% EtOH (two 2-min washes), washed with 40% EtOH (one 2-min wash), and rinsed with water for 30 s. After drying, the ^125^I-labeled sections were exposed to a BAS imaging plate (Fuji Film) overnight. Autoradiographic images were obtained using a BAS5000 scanner system (Fuji Film). After autoradiographic examination, the same sections were immunostained using an amyloid β-protein immunohistochemical staining kit (Wako Pure Chemical Industries, Ltd.).

## Results and Discussion

### Chemistry

The key step in the formation of the pyridyl benzofuran backbone was accomplished with a Suzuki coupling reaction (Figure 8) [27]. Suzuki coupling afforded the desired compounds **1**–**3** in yields of 31.5, 38.0, and 37.2%, respectively. The bromo compounds (**1**–**3**) were then reacted with bis(tributyltin) using Pd(0) as the catalyst and the corresponding tributyltin derivatives (**4**–**6**) were obtained in yields of 24.6, 28.4, and 25.1%, respectively. These tributyltin derivatives were readily reacted with iodine in chloroform at room temperature to give iodo derivatives (**7**–**9**) in yields of 52.0, 65.1, and 45.6%, respectively. Furthermore, these tributyltin derivatives can be also used as the starting materials for radioiodination in the preparation of [^125^I]**7**, [^123^I/^125^I]**8**, and [^125^I]**9**. Novel radioiodinated pyridyl benzofuran derivatives were obtained by an iododestannylation reaction using hydrogen peroxide as the oxidant (Figure 9). It was anticipated that the no-carrier-added preparation would result in a final product bearing a theoretical specific activity similar to that of ^125^I (81.4 TBq/mmol). The radiochemical identities of the radioiodinated ligands were verified by co-injection with the nonradioactive compounds by their HPLC profiles. [^125^I]**7**, [^123^I/^125^I]**8**, and [^125^I]**9** were obtained in 61–89% radiochemical yield with a radiochemical purity of >99% after purification by HPLC.

### In Vitro Binding to Aβ(1–42) Aggregates

Initial screening of the affinity of iodinated pyridyl benzofuran derivatives was carried out with Aβ(1–42) aggregates, using [^125^I]IMPY as the competing radioligand [22], [28]. These derivatives inhibited the binding of [^125^I]IMPY with *K_i_* values in the nanomolar range (*K_i_ = *2.4–10.3 nM), indicating that they had excellent affinity for Aβ(1–42) aggregates (Table 1). We have reported previously that ^18^F-labeled pyridyl benzofuran derivatives showed high affinity to Aβ(1–42) aggregates at *K*_i_ values from 2.4 to 3.9 nM, indicating that the pyridyl benzofuran core structure had considerable tolerance for structural modification with nucleophilic groups (NH_2_, NHMe, NMe_2_). The *K*_i_ values of IMPY and PIB as controls were 10.5 and 9.0 nM, respectively (Table 1). The affinity of iodinated pyridyl benzofuran derivatives was close to that of these known Aβ imaging tracers, suggesting that these derivatives possess enough affinity to image Aβ aggregates in vivo.

**Table 1:** Inhibition Constants for the Binding of [^125^I]IMPY to Aβ(1–42) Aggregates.

Compound	*K*_i_ (nM)^a^
**7**	10.3±1.48
**8**	2.94±0.22
**9**	2.36±0.53
IMPY^b^	10.5±1.05
PIB^b^	9.00±1.31

a Values are the means ± standard errors of the mean of three independent determinations.

b Data from ref. [24].

### In Vitro and in Vivo Stability in Mouse Plasma

**Figure 1:**
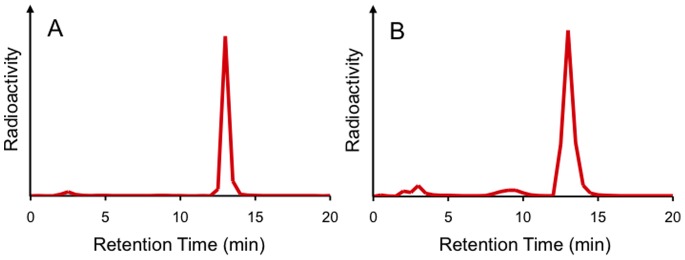
RP-HPLC profiles of [^125^I]8 in mouse plasma before (A) and after incubation for 1 h (B) at 37°C.

**Figure 2:**
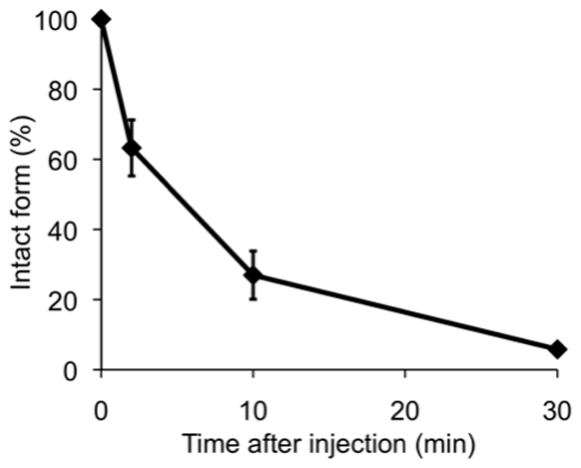
Percentage of the intact form in plasma after injection of [^125^I]8 into mice (n = 3). Time-activity curves obtained from RP-HPLC of plasma samples at 2, 5, and 30 min after injection of [^125^I]**8** into mice.

We determined the in vitro and in vivo stability of [^125^I]**8** in mouse plasma. When incubated [^125^I]**8** in mouse plasma for 1 h, 77.4% of radioactivity derived from [^125^I]**8** existed as an intact form (Figure 1), indicating that it should be relatively stable in mouse plasma. Conversely, as shown in Figure 2, when radioactivity was analyzed in mouse plasma by RP-HPLC after injection of [^125^I]**8** into mice, we found conversion of [^125^I]**8** to several different chemical forms. A percent of the intact form decreased with time and reached 20.7% at 30 min postinjection. This profile was comparable to that of [^11^C]PIB in mice reported previously [29], suggesting that [^123/125^I]**8** has sufficient in vivo stability for clinical trials. The difference in the stability of [^125^I]**8** between in vitro and in vivo may be attributable to radiometabolites produced by the in vivo metabolism of [^125^I]**8** in organs such as the liver and kidney.

### In Vivo Biodistribution in Normal Mice

**Figure 3:**
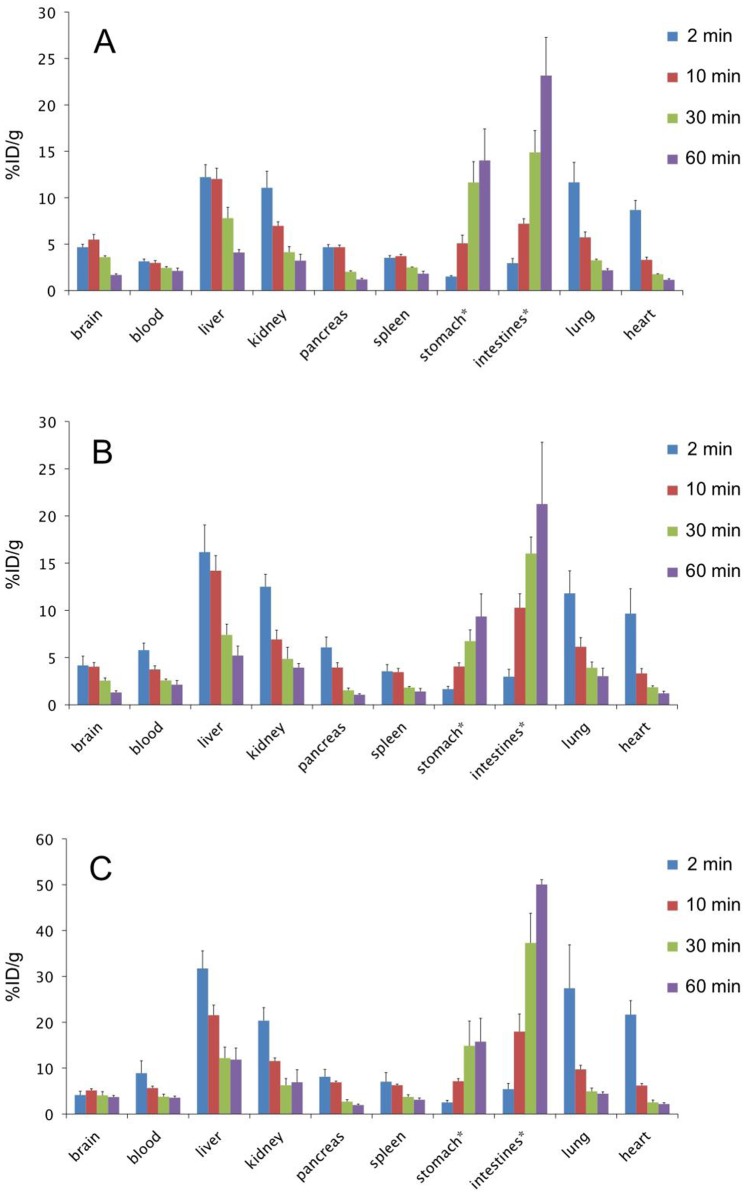
Biodistribution of radioactivity after injection of [^125^I]7 (A), [^125^I]8 (B), and [^125^I]9 (C) in normal mice. Values are expressed as the mean %injected dose/g SD in groups of five animals for each time point.

**Figure 4:**
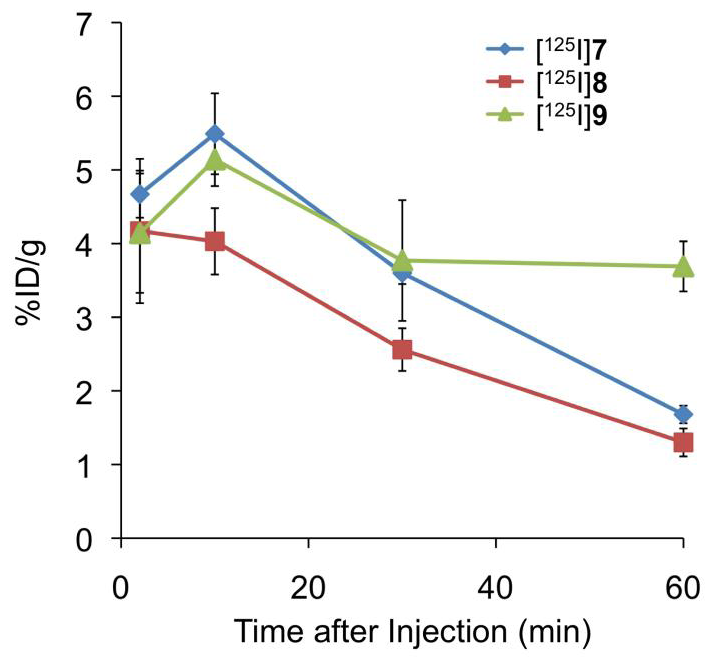
Comparison of brain uptake of three radioiodinated pyridyl benzofuran derivatives in normal mice.

We determined the biodistribution of radioactivity after an intravenous injection of [^125^I]**7**, [^125^I]**8**, and [^125^I]**9** in healthy mice (Figure 3). To define an acceptable PET or SPECT probe for imaging Aβ plaques in vivo, several common criteria have been set, such as: 1) high affinity for Aβ aggregates (*K*_i_ ≤20 nM); 2) a sufficient amount of the agent entering the brain and non-specifically bound agent cleared from the brain; high initial uptake >0.5%ID/g at 2 min postinjection and quick washout from the brain (<30% of initial uptake remaining in the brain) [6], [20] To directly compare the uptake and washout of three radioiodinated probes, a combined plot is presented in Figure 4. These ^125^I-labeled derivatives penetrated the blood-brain barrier (BBB) soon after the injection (4.14–4.67%ID/g at 2 min postinjection), and the radioactivity cleared with time from the brain (1.30–3.69%ID/g at 60 min postinjection). [^125^I]**8** peaked at 2 min postinjection, while [^125^I]**7** and [^125^I]**9** peaked at 10 min postinjection. Generally, the brain_2min_/brain_60 min_ ratio has been used to compare the rate of washout from the brain. The brain_2min_/brain_60 min_ ratios of [^125^I]**7**, [^125^I]**8**, and [^125^I]**9** were 1.30, 3.21 and 1.12, respectively. The results in normal mice showed that the monomethylated pyridyl benzofuran derivative [^125^I]**8**, the brain_2min_/brain_60 min_ ratio of which was 3.21, exhibited not only high initial uptake in the brain but also quick washout from the brain over time, which are highly desirable properties for Aβ imaging probes (Figure 3). We have previously reported the development of radioiodinated phenylbenzofuran derivatives as SPECT imaging agent [30]. Although these derivatives displayed high uptake in normal mouse brain, the radioactivity washed out slowly, suggesting high non-specific binding in vivo. While the brain_2min_/brain_60 min_ ratio of the radioiodinated phenylbenzofuran derivatives ranged from 0.47 to 0.65, the low brain_2min_/brain_60 min_ ratio of the radioiodinated pyridyl benzofuran derivatives (1.12–3.21) indicated much better pharmacokinetics in the brain than the phenylbenzofuran derivatives. Furthermore, the brain_2min_/brain_60 min_ ratio of [^125^I]**8** (3.2) was close to that of [^18^F]AV-45 (3.8) [31], which is a PET imaging probe approved by the FDA, indicating that [^125^I]**8** shows excellent pharmacokinetics in the brain sufficient to apply to clinical studies. We have reported previously that the brain_2min_/brain_60 min_ ratio of ^18^F-labeled pyridyl benzofuran derivatives was 2.11–2.34, indicating that [^125^I]**8** provided a better profile than ^18^F-labeled pyridyl benzofuran derivatives. The favorable in vivo pharmacokinetics of [^125^I]**8** were achieved by substitution of the phenyl group with a pyridyl group, which resulted in a less lipophilic compound. Since lipophilicity is an important factor affecting the uptake of a compound into the brain, it may be associated with the favorable pharmacokinetics of [^125^I]**8** in the brain.

Radioactivity also distributed to several other organs, such as the liver, kidneys, stomach, and intestines, in addition to accumulating in the brain. The liver and kidneys showed initially high uptake and washout with time while the stomach and intestines showed an accumulation of radioactivity over time (Figure 3).

### In Vivo Evaluation with Tg2576 mice

**Figure 5:**
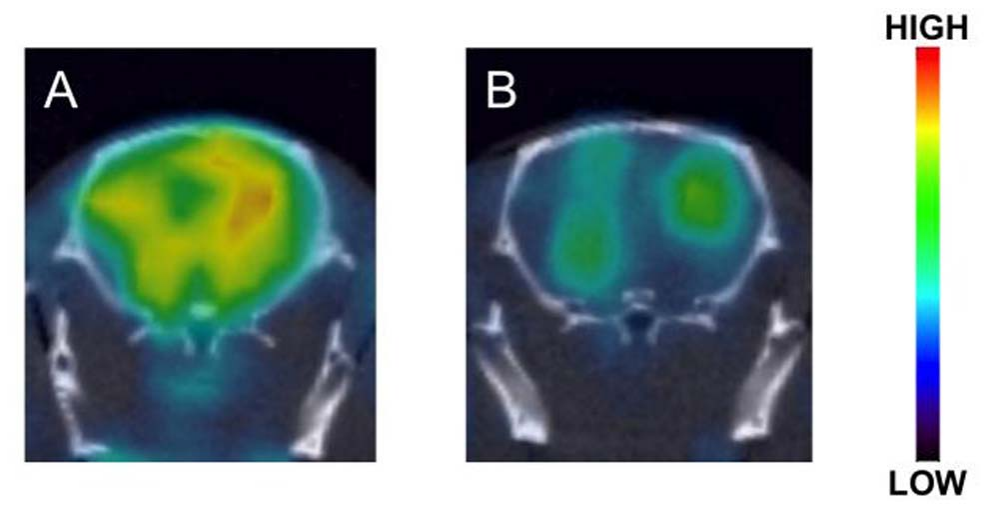
Brain SPECT images after injection of [^123^I]8 into a Tg2576 mouse (A) and a wild-type mouse (B).

To prove the feasibility of Aβ imaging in vivo, SPECT/CT was performed in Tg2576 transgenic mice and wild-type mice after the intravenous injection of [^123^I]**8**. Tg2576 transgenic mice have been widely used for in vitro and in vivo evaluations of Aβ imaging agents because of marked Aβ deposition in the brain [32]. In transverse SPECT images, a clear difference in the accumulation of radioactivity in the brain was observed between Tg2576 and wild-type mice (Figure 5). The mean FR/CE ratios were 1.34±0.07 (average ± SD) in Tg2576 mice (n = 3) and 1.01±0.03 (average ± SD) in wild-type mice (n = 3), the difference being significant (*p* = 0.012). Although further quantitative studies are necessary in the future, the statistical analyses appear to reflect the difference of radioactivity accumulation on the SPECT images between Tg2576 and wild-type mice.

**Figure 6:**
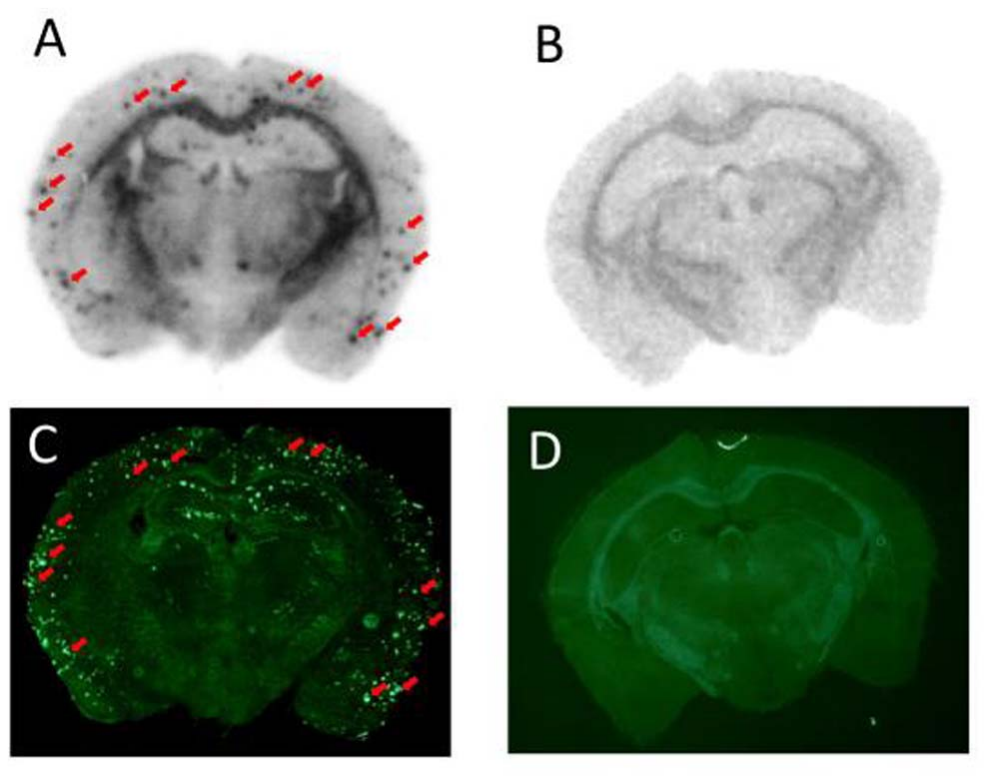
Ex vivo autoradiograms of brain sections after injection of [^123^I]8 into a Tg2576 mouse (A) and a wild-type mouse (B). Fluorescent staining of the same brain section (A and B) with thioflavin S is shown in Figures C and D, respectively.

To further characterize the specific binding of [^123^I]**8** to Aβ plaques in brains, the brains were removed, frozen, and sectioned immediately after the SPECT/CT study for ex vivo autoradiography (Figure 6). Autoradiographic images showed extensive labeling of Aβ plaques in the brains of Tg2576 mice (Figure 6A) but not the wild-type control (Figure 6B). The labeling of Aβ plaques was confirmed by co-staining the same sections with thioflavin-S, a pathological dye commonly used for amyloid staining (Figure 6C). These results suggested that [^123^I]**8** penetrated the intact BBB and bound to cerebral Aβ plaques strongly. Many Aβ-targeting tracers for SPECT reported previously did not penetrate the BBB or once they entered the brain, the nonspecific binding was so high that the agents could not provide a specific signal for Aβ plaques, despite their high binding affinity. To our knowledge, this is the first report that Aβ plaques in Tg2576 mouse brain were successfully imaged in vivo with a small-animal SPECT. These results also suggested the feasibility of in vivo Aβ imaging with [^123^I]**8** in the brains of AD patients.

### Autoradiography of Postmortem AD Brain Sections

**Figure 7:**
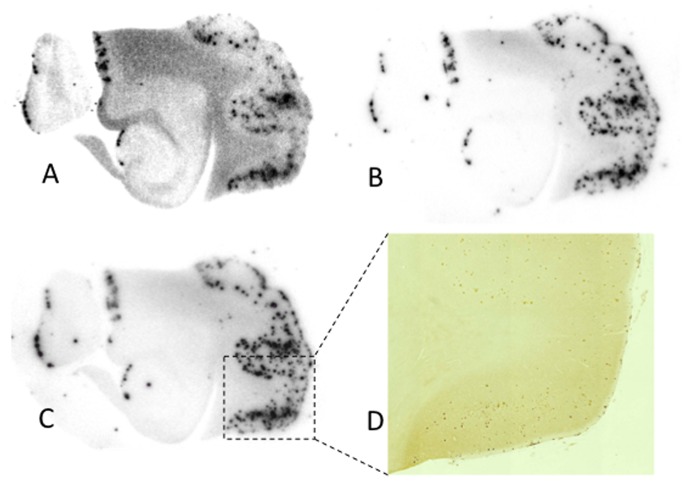
In vitro autoradiograms of sections of the AD brain labeled with [^125^I]7 (A), [^125^I]8 (B), and [^125^I]9 (C). The Aβ plaques were confirmed by immunostaining (D).

To evaluate the binding of [^125^I]**8** to Aβ plaques accumulated in human brains, in vitro autoradiography in postmortem AD brain tissue sections was performed. A previous study reported the conformation of Aβ plaques in Tg2576 mice to be different from that in AD brains [33]. It is therefore very important to test the affinity for Aβ plaques in AD brains in addition to evaluations using Tg2576 mice. When the AD brain sections were incubated with ^125^I-labeled pyridyl benzofurans, autoradiographic images revealed excellent visualization of Aβ plaques with minimal background noise (Figure 7). No marked difference in the accumulation of radioactivity in AD brain sections was observed between [^125^I]**8** and [^125^I]**9** (Figure 7B and 7C). However, [^125^I]**7** showed nonspecific binding in white matter in the brain, while it labeled Aβ plaques (Figure 7A). The spots of radioactivity derived from [^125^I]**8** in the cortex were confirmed by immunostaining the same brain sections with Aβ antibody (Figure 7D). These results indicated that [^123^I]**8** may be applied to the in vivo imaging of Aβ plaques in AD brains, and deserves further investigation as a promising SPECT imaging agent for the diagnosis of AD. Although a similar autoradiographic pattern was observed for both [^125^I]**8** and [^125^I]**9**, [^125^I]**7** displayed nonspecific accumulation in white matter with the binding to Aβ plaques. In the binding assay, [^125^I]**8** (*K*_i_ = 2.94 nM) and [^125^I]**9** (*K*_i_ = 2.36 nM) showed higher affinity for Aβ aggregates than [^125^I]**7** (*K*_i_ = 10.3 nM) (Table 1). Therefore, the difference in the autoradiographic study may be attributable to the affinity of the compounds.

**Figure 8:**
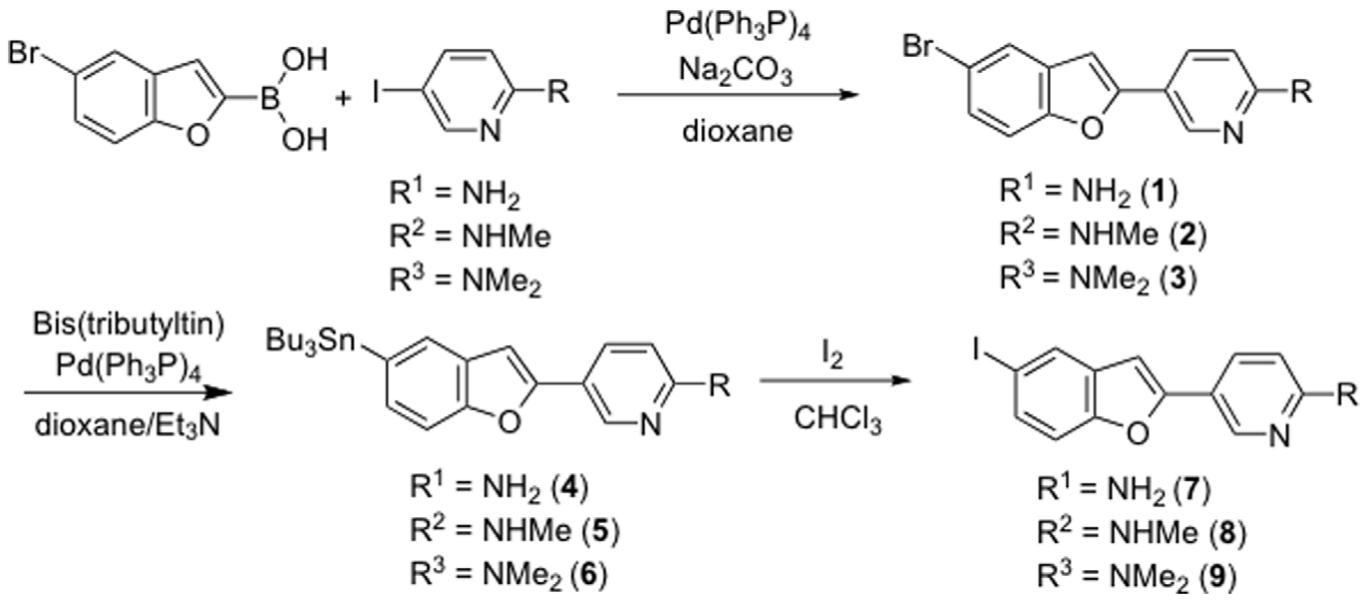
Scheme 1. Synthesis of iodinated pyridyl benzofuran derivatives.

**Figure 9:**
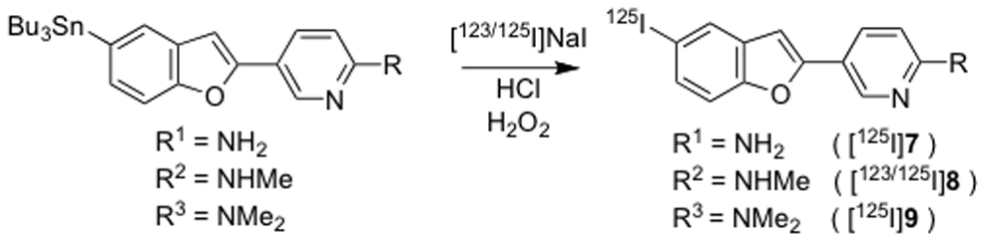
Scheme 2. Radiosynthesis of iodinated pyridyl benzofuran derivatives.

## Conclusion

On the basis of our previous results, we developed three novel iodinated pyridyl benzofuran ligands for imaging Aβ plaques in the brain. These ligands showed high affinity for Aβ aggregates in vitro and for Aβ plaques in sections of an autopsied AD brain. In biodistribution experiments using normal mice, [^125^I]**8** displayed good uptake into and fast washout from the brain. In addition, a SPECT study and ex vivo autoradiograms of brain sections from Tg2576 mice after the injection of [^123^I]**8** showed selective binding of Aβ plaques with little nonspecific binding. The results suggest that [^123^I]**8** should be further investigated as a potentially useful SPECT imaging agent for cerebral Aβ plaques in the AD brain.
